# Multi-Scale Effects of Nestling Diet on Breeding Performance in a Terrestrial Top Predator Inferred from Stable Isotope Analysis

**DOI:** 10.1371/journal.pone.0095320

**Published:** 2014-04-17

**Authors:** Jaime Resano-Mayor, Antonio Hernández-Matías, Joan Real, Marcos Moleón, Francesc Parés, Richard Inger, Stuart Bearhop

**Affiliations:** 1 Equip de Biologia de la Conservació, Departament de Biologia Animal, Universitat de Barcelona, Barcelona, Catalonia, Spain; 2 Departamento de Biología Aplicada, Universidad Miguel Hernández, Orihuela, Alicante, Spain; 3 Environment and Sustainability Institute, University of Exeter, Cornwall Campus, Penryn, Cornwall, United Kingdom; 4 Centre for Ecology and Conservation, School of Biosciences, University of Exeter, Cornwall Campus, Penryn, Cornwall, United Kingdom; University of Lleida, Spain

## Abstract

Inter-individual diet variation within populations is likely to have important ecological and evolutionary implications. The diet-fitness relationships at the individual level and the emerging population processes are, however, poorly understood for most avian predators inhabiting complex terrestrial ecosystems. In this study, we use an isotopic approach to assess the trophic ecology of nestlings in a long-lived raptor, the Bonelli’s eagle *Aquila fasciata*, and investigate whether nestling dietary breath and main prey consumption can affect the species’ reproductive performance at two spatial scales: territories within populations and populations over a large geographic area. At the territory level, those breeding pairs whose nestlings consumed similar diets to the overall population (i.e. moderate consumption of preferred prey, but complemented by alternative prey categories) or those disproportionally consuming preferred prey were more likely to fledge two chicks. An increase in the diet diversity, however, related negatively with productivity. The age and replacements of breeding pair members had also an influence on productivity, with more fledglings associated to adult pairs with few replacements, as expected in long-lived species. At the population level, mean productivity was higher in those population-years with lower dietary breadth and higher diet similarity among territories, which was related to an overall higher consumption of preferred prey. Thus, we revealed a correspondence in diet-fitness relationships at two spatial scales: territories and populations. We suggest that stable isotope analyses may be a powerful tool to monitor the diet of terrestrial avian predators on large spatio-temporal scales, which could serve to detect potential changes in the availability of those prey on which predators depend for breeding. We encourage ecologists and evolutionary and conservation biologists concerned with the multi-scale fitness consequences of inter-individual variation in resource use to employ similar stable isotope-based approaches, which can be successfully applied to complex ecosystems such as the Mediterranean.

## Introduction

The trophic niche of a species refers to the range of food sources it uses and is a key component of the n-dimensional hypervolume niche concept [1]. Classical models based on the optimal foraging theory assume that individuals within populations respond similarly to spatial and temporal heterogeneity in resource availability [2]–[4]. According to this theory, individual consumers are expected to take preferred resources when food availability is high, or expand their food range by adding suboptimal resources when resource availability lowers [5]–[7]. More recently, variations in trophic resource use among individuals within a population have been highlighted to be a widespread phenomenon in nature, what may result from a broad range of mechanisms [8]. Apart from sex, age or individual’s phenotype, individual diet variation may arise from their differences in dominance, experience or foraging ability [8]–[12]. Consequently, both extrinsic ecological factors and intrinsic organismal traits may generate a dietary spectrum within the population.

Despite the increasing understanding of the causes of individual diet variation within animal populations, its implications in terms of individual fitness are still poorly understood. Differences in trophic resource use among individuals are likely to affect their energy income and, therefore, to have an impact on fitness [13]–[15]. In this regard, individual diet variation has been shown to differently influence breeding success in several avian species, an issue particularly studied in seabirds [16]–[19]. For instance, individuals with lower diet diversity may increase their breeding success provided that low trophic diversity is the result of specialization on preferred prey [16], [20]. The superior fitness of individuals with specialized diets, however, is not a universal pattern, and numerous studies have documented the fitness benefits of a generalized diet [19], [21]. Therefore, the fitness consequences of individual dietary variation are not always easy to predict as different feeding strategies may be advantageous for different species and/or ecological scenarios, or even between different individuals within a population, depending on multiple factors like the nutritional quality of food, the physiology of the consumer, or the spatial and temporal availability of prey [21]–[24].

Several important drivers of the structure and dynamics of populations and communities, such as intraspecific competition, predation risk or parasitism, are linked to individual’s resource use. Consequently, theoretical studies have highlighted the importance of intraspecific diet variation in shaping populations and communities [25]–[27]. In this regard, several studies have linked lower dietary diversity with higher breeding success at the population level, what has been explained by a higher consumption of preferred prey in those populations with lower diet diversity [28]–[30]. Conversely, populations exposed to heterogeneous landscapes or changing environmental conditions (e.g. food availability) can perform better if individuals within the population diversify in food resource use [8]. In fact, the correspondence between both scales (i.e. intra-population resource use and population processes) remains largely unknown in most natural systems so further studies are required to investigate how the relationship between diet and fitness at the individual level translates to the population scale.

Stable isotope analyse (SIA) of carbon (δ^13^C) and nitrogen (δ^15^N) has been increasingly used by animal ecologists to assess individual resource use and intra-population niche partitioning [31]–[33], as it provides insightful dietary information at the individual level difficult to obtain by conventional procedures. In particular, the isotopic composition in metabolically inert tissues (e.g. feathers), represents consumer’s diet at the time of deposition (e.g. feather growth), so SIA are a powerful tool to assess animal (e.g. avian) temporal and spatial dietary information at both the individual and population levels [34], [35]. Consumer isotopic data delineated in δ-space (e.g. δ^13^C-δ^15^N bi-plot), has been termed the “isotopic niche” [32], and is thought to be closely aligned with the true trophic niche. Recently developed metrics based on δ-space also allow for a species’ trophic structure and diet diversity to be quantified not only at the individual, but also at the population and community levels [36], [37]. Complementarily, the use of isotopic mixing models allow for individual diet reconstructions by converting isotopic data of consumers and main food resources into dietary proportions (p-space) [38]–[40], that can be translated into niche-width metrics commonly used by ecologists [41]. Surprisingly, the use of SIA to assess the trophic ecology of terrestrial avian top predators, either at the territory or the population levels, has rarely been done (but see [24], [42]).

In this study, we use an isotopic approach to assess the trophic ecology of a long-lived raptor, the Bonelli’s eagle *Aquila fasciata*, to explore the relationship between nestling diet and reproductive performance at both the territorial and population scales. Bonelli’s eagle is a suitable model species because it shows marked intra- and inter-population demographic variations across its western European distribution range [43]. Moreover, the marked population decline occurred in this area in recent decades has been partly related to habitat degradation and main prey scarcity [44]. Here, both European rabbits *Oryctolagus cuniculus* (mostly) and red-legged partridges *Alectoris rufa* have been suggested to be optimal prey for Bonelli’s eagle, so that they are preferentially consumed wherever they are abundant, and their consumption reduces eagle’s diet diversity [45]–[49]. The dietary variation among territories is however substantial, and is thought to be influenced by habitat heterogeneity linked to crashes in rabbit numbers around two decades ago due to outbreaks of rabbit haemorrhagic disease, which indirectly impacted on the abundance of partridges and possibly other prey [46], [48], [50], [51]. In the post-disease period, the average consumption of rabbits and partridges has been relatively low in most populations of western Europe, particularly in those located in the north of the Iberian Peninsula and France [48], what could reveal unprecedented effects of eagles’ diet on fitness components such as breeding success (see [46]).

The specific objectives of this study were to i) describe the isotopic niche width and structure (as a proxy of trophic niche) of Bonelli’s eagle nestlings in three populations of western Europe, ii) estimate nestling prey consumption at the intra-population (i.e. territory) level using isotopic mixing models, and iii) test the relationship between the dietary estimates and isotopic niche metrics with breeding success at both the territory and population levels. Our main prediction is that those territories and populations where nestlings have narrower trophic niches, what is expected to occur where nestling diets largely rely on rabbits and/or partridges, will perform better in terms of breeding success.

## Materials and Methods

### Ethics Statement

Both the monitoring of breeding pairs and sample collection of Bonelli’s eagles were conducted to conform to the legal requirements of competent organisms. Permission to monitor breeding activity and to access nests in order to handle and sample eagle’s nestlings and prey remains was granted by the CRBPO (Centre de Recherches sur la Biologie des Populations d’Oiseaux) that validates the banding program delegated by the Ministère de l’Écologie, du Développement durable et de l’Énergie (French Government) in France, by the “Servei de Biodiversitat i Protecció dels Animals” (Generalitat de Catalunya) in Catalonia, and by “Consejería de Medio Ambiente” (Junta de Andalucía) in Andalusia. To avoid disturbance, breeding monitoring observations were always carried out away from nests by using 10x binoculars and 20–60x spotting scopes.

### Study Area

**Figure 1:**
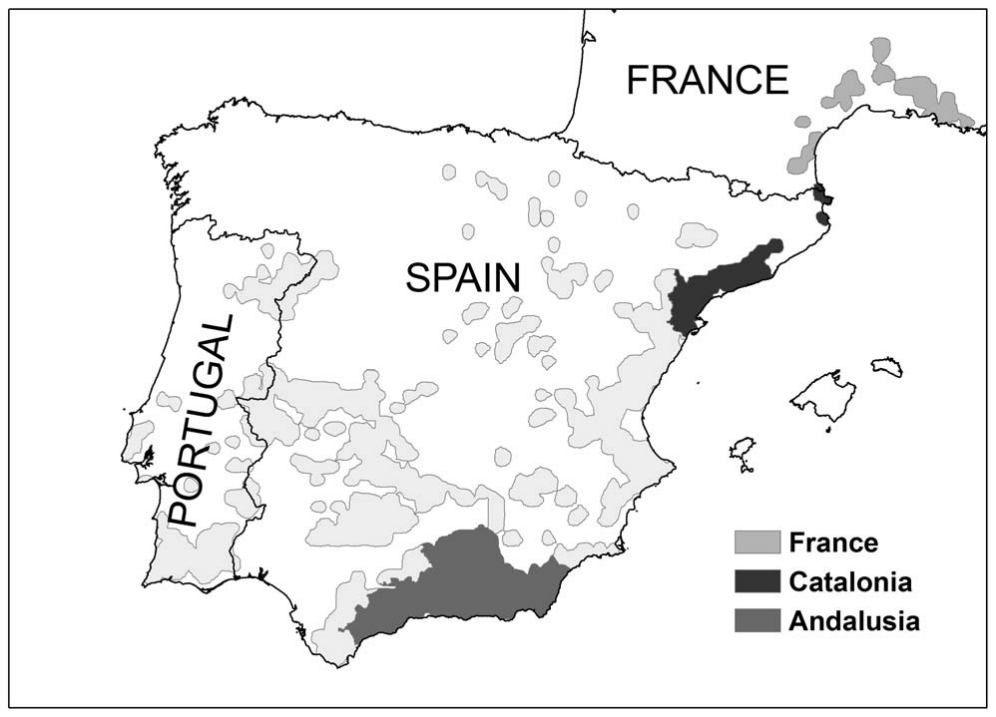
Distribution area (shaded polygons) of Bonelli’s eagle in western Europe (modified from [43]). In darker grey we show the monitored populations, north to south, southern France, Catalonia and Andalusia (see legend).

From 2008 to 2011 we monitored the main vital rates in 131 territorial Bonelli’s eagle pairs located in three populations across the species’ western European range, north to south, Provence and Languedoc-Roussillon (43°58**′**N, 03°20**′**E; southeast France; *n* = 30 and 31 pairs in 2010 and 2011, respectively), Catalonia (41°20**′**N, 01°32**′**E; northeast Spain; *n* = 52, 40, 44 and 45 in 2008, 2009, 2010 and 2011, respectively) and Andalusia (37°76**′**N, 03°85**′**W; southeast Spain; *n* = 45 in 2011) (Figure 1). The population in France had a breeding density of 0.29 territorial pairs/100 km^2^, it showed a mean productivity of 0.98 fledglings/pair, an adult survival of 0.880, and the mean percentage of non-adult birds in territorial pairs was 8.39. The population in Catalonia had a breeding density of 0.64, it showed slightly higher values of productivity (1.13) and adult survival (0.889), and the mean percentage of non-adult birds was 10.81. The population in Andalusia had a breeding density of 0.85, it showed the highest values of productivity (1.23) and adult survival (0.926), and the mean percentage of non-adult birds was 3.86 [43]. All breeding nests were located on cliffs, and territories varied markedly with respect to habitat features, prey abundances or human activity [44].

### Data Collection

To assess the breeding success and productivity of monitored territorial pairs, known breeding areas were yearly visited a minimum of five days during the whole breeding period. Between January and March we checked the presence of territorial birds and breeding activity (i.e. incubation behaviour). In late March and April, occupied nests were checked to detect the presence, number, and age of nestlings, which was estimated by feather development and backdating from laying date [52]. Nestlings at the age of ≥50 days old were assumed to have fledged successfully [53].

For each territorial pair, individual turnover events or replacements (i.e. if the same individuals/pairs occupied the same territories across years) were estimated by comparing the plumage-ages of the male and the female in two consecutive years [54]. Each pair was classified as adult (both individuals with an adult plumage) or non-adult (at least one individual with a non-adult plumage) [55].

Once nestlings were on average 35–40 days old, we accessed breeding nests with the assistance of experienced climbers to sample four mantle feathers from each chick for SIA purposes. Nestlings were sampled in a subset of the monitored territories, involving 21 different breeding territories in France (*n* = 20 and 12 in 2010 and 2011, respectively), 38 in Catalonia (*n* = 20, 17, 25, and 24 in 2008, 2009, 2010 and 2011, respectively) and 12 in Andalusia 2011. The isotopic composition of sampled feathers reflects nestling diet during tissue development and was used to estimate both the isotopic niche metrics at the population level and the proportional contribution of main prey categories to nestling diets at the territory-year level (see below).

To characterize main prey isotopic values we obtained samples from the three studied populations by collecting a small piece of muscle from carcasses found at the nests at the same time as chick feathers were sampled. Prey tissue collections were supplemented with dead individuals collected in the surroundings of eagles’ breeding territories in Catalonia (see [42]). We did not find any evidence of prey isotopic differences among the three populations when comparing the main prey categories, i.e. rabbits, partridges and pigeons. Therefore, we combined all samples of each prey group to calculate mean ± SD prey isotopic values for the whole study area.

### Stable Isotope Analyses

The isotopic ratios of Carbon (^13^C:^12^C) and Nitrogen (^15^N:^14^N) were measured for both nestling feathers and prey muscle samples following the procedure described in [42]. Subsamples of approximately 0.35 mg of feathers and 0.32 mg of muscle were loaded in tin recipients and crimped for combustion. All isotopic measurements were conducted at the “Centres Científics i Tecnològics, Universitat de Barcelona”.

Stable isotope values are reported following the conventional δ notation, where δ^13^C or δ^15^N = [(R_sample_/R_standard_) –1]*1000 (‰), and R is the corresponding ratio ^13^C:^12^C or ^15^N:^14^N. International R_standards_ were Pee Dee Belemnite (PDB) for δ^13^C and atmospheric nitrogen (AIR) for δ^15^N. The measurement precisions were ≤0.15‰ and ≤0.25‰ for δ^13^C and δ^15^N, respectively.

### Population Isotopic Niche Metrics

Our population approach was based on each population-year sampled (n = 7; France 2010–2011, Catalonia 2008–2011 and Andalusia 2011). To address Bonelli’s eagle nestling trophic structure at the population-year level we used the isotopic metrics originally proposed by Layman et al. [36], and recently extended by Jackson et al. [37]. Each territory-year was a single observation; in territories with two chicks, we calculated mean isotopic values of siblings because they showed similar isotopic values (see [56]). The following isotopic niche metrics were calculated based on the δ^13^C-δ^15^N bi-plot (i.e. δ-space) as described in [37]: δ^13^C range (CR_b_) and δ^15^N range (NR_b_) to assess the total carbon and nitrogen ranges in the consumed prey; mean distance to centroid (CD_b_) as a measure of population trophic diversity; and standard deviation of nearest neighbour distance (SDNND_b_) to infer population trophic evenness. All these metrics were bootstrapped (‘_b_’; *n* = 10000) based on the minimum number of territories (*n* = 12) in the data set of population-years, which allows comparisons among population-years despite different territorial sample sizes [57]. Finally, we calculated the corrected standard ellipse area (SEA_c_) to estimate the isotopic niche width of each population-year. The SEA_c_ measures the core isotopic niche area (ca. 40% of the data) and corrects for bias associated with small sample sizes [37].

### Nestling Prey Consumption Estimates

**Figure 2:**
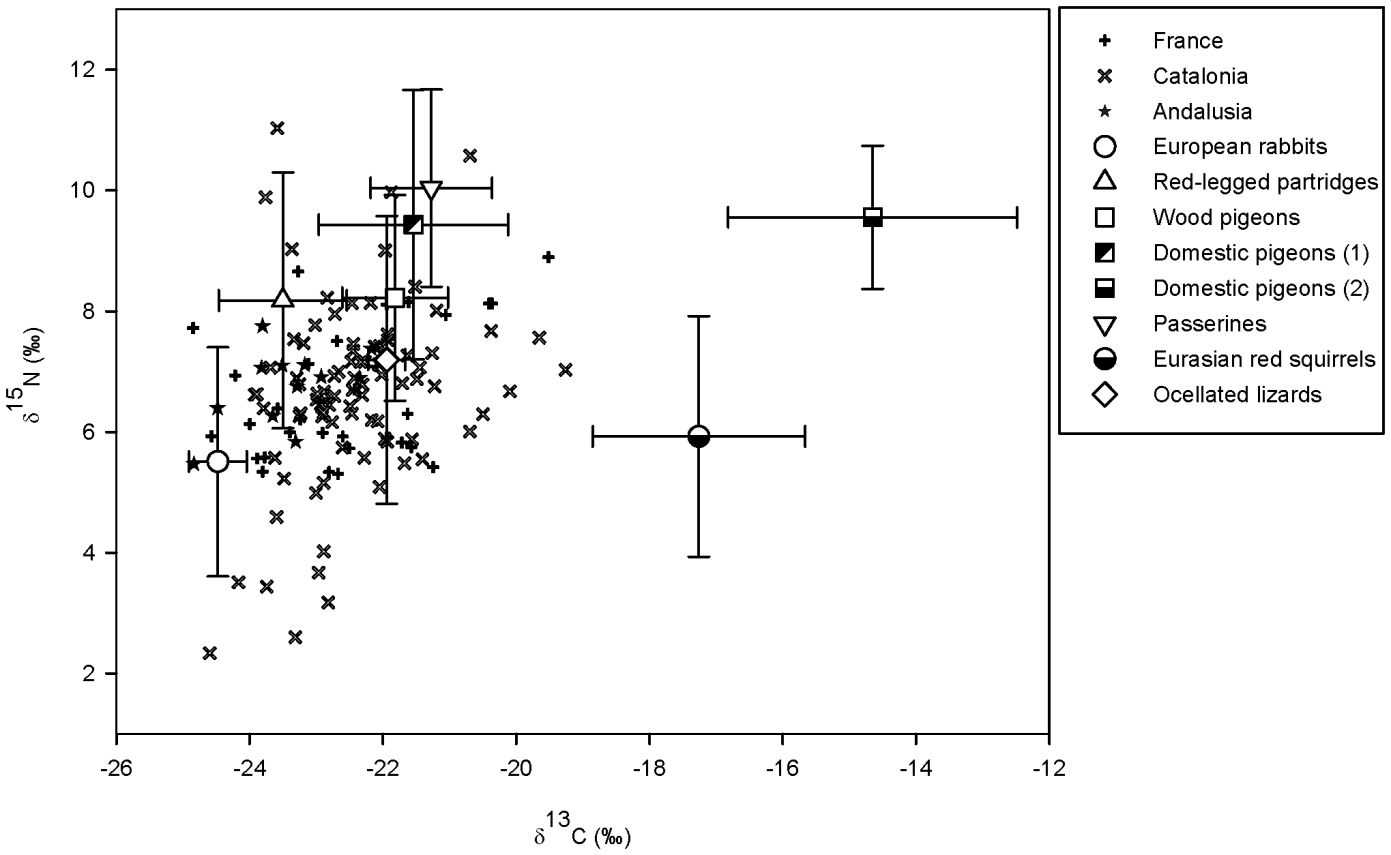
Stable carbon (δ^13^C) and nitrogen (δ^15^N) isotope values (‰) in Bonelli’s eagle nestlings and their main prey. Prey isotopic values were corrected for trophic discrimination factors (i.e. 2.1 and 2.7‰ for δ^13^C and δ^15^N, respectively; [59]). Single data points represent nestlings of each territory-year, with different symbols for France, Catalonia and Andalusia. Prey categories (mean ±95% SD) included: European rabbits, red-legged partridges, wood pigeons, domestic pigeons wildly foraging in crops (1), domestic pigeons from dovecotes and fed with corn (2), passerines, Eurasian red squirrels, and ocellated lizards (see figure legend).

We used the Bayesian mixing model SIAR (Stable Isotope Analysis in R; [40], [58]) to estimate the relative contribution of main prey categories to nestling diets at the territory-year level. Main prey categories included into SIAR were European rabbits, red-legged partridges, wood pigeons *Columba palumbus*, domestic pigeons *C. livia* dom., passerines (Corvidae, Sturnidae and Turdidae), Eurasian red squirrels *Sciurus vulgaris*, and ocellated lizards *Timon lepidus* [48]. Based on δ^13^C values, domestic pigeons were divided in two categories to account for the marked isotopic differences observed between individuals foraging on crops (i.e. lower δ^13^C) and those associated with dovecotes and fed with corn *Zea mays* (i.e. higher δ^13^C) (see Table S1 for prey isotopic values). The δ^13^C-δ^15^N bi-plot with nestling and main prey isotopic values corrected for trophic discrimination factors (TDFs) is shown in Figure 2. We used TDFs values of 2.1‰ ±0.08 for δ^13^C and 2.7‰ ±0.5 for δ^15^N, calculated from feathers of peregrine falcons *Falco peregrinus* fed on muscle of Japanese quail *Coturnix japonica* [59]. Mean prey consumption estimates from SIAR were selected for subsequent analyses. To investigate the sensitivity of prey consumption estimates to inaccuracy in our TDFs, we varied 1‰ those values used for δ^13^C (1.6–2.6‰) and those used for δ^15^N (2.2–3.2‰) by maintaining the SD of 0.08 and 0.5 for δ^13^C and δ^15^N, respectively [59]. On average, main prey consumption estimates varied less than 4% for the mean contributions to the diet. Therefore, we were confident on our previous results based on the TDFs obtained from the literature since even changes in 1‰ in the TDFs do not considerably affect our mean prey consumption estimates from SIAR.

### Data Analysis

Main prey consumption estimates from SIAR were used to assess nestlings’ diet diversity and prey consumption specificity at the territory-year level. The Shannon-Weaver index (H′) was used to calculate the diet diversity [60]. To estimate nestlings’ prey consumption specificity we used the proportional similarity index (PS_i_) [41], which measures the diet overlap between an individual (i.e. nestlings in a territory-year in our approach) and its population (i.e. mean diet in the whole set of territory-years sampled in the study area). PS_i_ tends towards 1 in those territories where nestling prey consumption is similar to the mean population diet, and is increasingly lower when prey consumption differs from the mean diet. In order to enhance the interpretation of our results, we also assessed the relationship between H′ and PS_i_. Moreover, we tested the existence of preferred prey in our study area by relating mean consumption of each prey category and H′ at the territory level through Spearman rank correlation tests [49], as higher consumption of preferred prey has been suggested to be inversely related with predator’s H′ [6].

To test the diet-fitness relationship at the territory-year level we applied Generalized Linear Mixed Models (GLMMs), which allowed accounting for the potential non-independence of clustered observations from the same territories, years and populations. Due to limitations imposed by the isotopic analysis (i.e. nestlings can only be sampled if breeding pairs are successful in hatching and rearing chicks), our territorial diet estimates only included successful breeding pairs. In our study area, spatial autocorrelation in productivity is absent between populations [43] and, therefore, we included territory and year nested by population as random categorical factors. In this analysis, productivity of breeding successful pairs (i.e. probability of producing two chicks instead of one) was modeled as a binomial response variable using a logit link function. Error distributions were assumed to be binomially distributed. We evaluated a set of models including the following explanatory variables: age of the breeding pair and mate replacement (categorical factors); consumption of rabbits, consumption of partridges, H′ and PS_i_ (continuous variables). We also included the interaction between H′ and PS_i_, and the quadratic effect of these two variables to test whether a parabolic trend fitted the model response (i.e. higher and lower values of either H′ or PS_i_ imply an advantage/disadvantage in terms of productivity compared with intermediate values of these variables) (see Table S2 for details on model parameters and their interactions). Ages of the breeding pair and mate replacements were included in all the models due to their potential influence on reproductive parameters [61], [62]. GLMMs were fitted using the lmer function from the lme4 package of R [63]. Model selection was based on Akaike’s Information Criterion adjusted for sample size (AIC_c_), computing the Akaike weights (AIC_cw_) to assess the probability that each candidate model was the best of the proposed set [64]. The goodness-of-fit of each model was estimated from marginal (R^2^_GLMM(m)_) and conditional (R^2^_GLMM(c)_) coefficients of determination, following [65]. The R^2^_GLMM(m)_ value shows the proportion of the variance in the raw data explained by the fixed effects only, while the R^2^_GLMM(c)_ value shows the proportion of the variance explained by the full model, including both fixed and random effects.

Regarding the diet-fitness relationship at the population-year level, we used Spearman rank correlation tests to assess for any relationship between mean productivity and either SEA_c_ or SDNND_b_. Mean productivity for each population-year was calculated either as the mean number of fledglings in the successful breeding pairs or the mean productivity in all monitored territorial pairs (i.e. successful breeders or not). In this second analysis we assumed that the SEA_c_ and the SDNND_b_ obtained from successful breeders were representative of the trophic niche structure in the whole population-year.

Statistical analyses were conducted using the R statistics platform (CRAN 2009) and SPSS (PASW 18.0).

## Results

### Population Isotopic Niche Metrics

**Figure 3:**
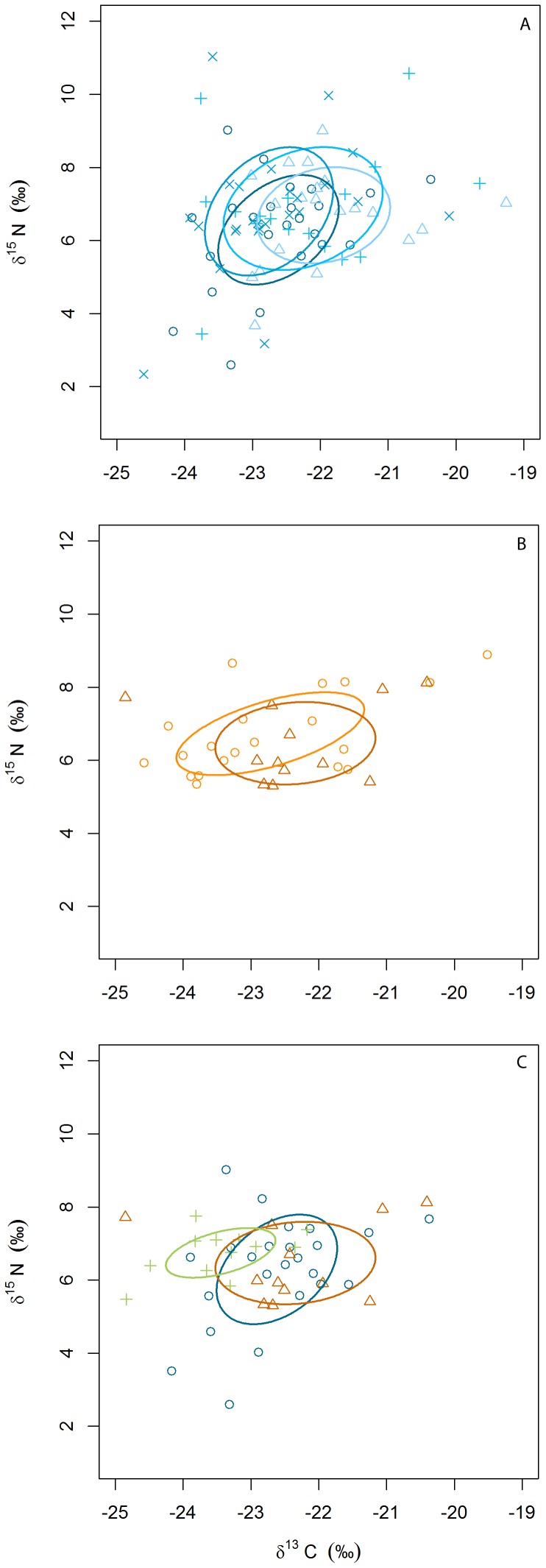
Corrected standard ellipse areas (SEA_c_) estimated from δ^13^C and δ^15^N values (‰) in Bonelli’s eagle nestlings. A) SEA_c_ in Catalonia 2008–2011 (light to dark blue), B) SEA_c_ in France 2010–2011 (light to dark orange), and C) SEA_c_ in France, Catalonia and Andalusia 2011 (dark orange, dark blue and green, respectively). Same symbols represent nestlings from the same population-year.

Overall, the isotopic values of Bonelli’s eagle nestlings ranged from −24.85 to −19.26‰ for δ^13^C (mean ± SD: −22.56±1.08‰; *n* = 130 nests), and from 2.34 to 11.03‰ for δ^15^N (6.65±1.36‰). France 2010 had the largest CR_b_, Catalonia 2009 the largest NR_b_, while Andalusia 2011 showed the lowest values for both CR_b_ and NR_b_. The CD_b_ and SDNND_b_ in Andalusia 2011 was substantially lower than all other population-years, suggesting higher trophic diversity and lower even distribution of nestling trophic niches in France and Catalonia if compared with Andalusia. Accordingly, the SEA_c_ in Andalusia 2011 was notably lower than any other population-year, being four times lower than Catalonia 2009, the highest SEA_c_ obtained in our study (Table 1; Figure 3).

**Table 1:** Isotopic niche metrics of Bonelli’s eagle nestlings at the population-year level.

Population-year	Mean δ^13^C	Mean δ^15^N	CR_b_	NR_b_	CD_b_	SDNND_b_	SEA_c_	*n*
France-2010	−22.71	6.73	3.95	3.09	1.47	0.49	4.01	20
France-2011	−22.34	6.47	3.46	2.68	1.28	0.50	4.15	12
Catalonia-2008	−21.95	6.69	2.88	3.97	1.29	0.49	3.99	20
Catalonia-2009	−22.25	6.87	3.38	5.29	1.56	0.64	5.98	17
Catalonia-2010	−22.76	6.80	2.99	5.82	1.43	0.74	4.92	25
Catalonia-2011	−22.62	6.29	2.76	4.72	1.35	0.51	3.86	24
Andalusia-2011	−23.45	6.75	2.32	1.94	0.81	0.29	1.49	12

Note: CR_b_ = δ^13^C range; NR_b_ = δ^15^N range; CD_b_ = mean centroid distance; SDNND_b_ = standard deviation of nearest neighbour distance; SEA_c_ = corrected standard ellipse area; *n* = number of sampled territories. All metrics except SEA_c_ were bootstrapped (‘_b_’; *n* = 10000).

### Prey Consumption Estimates

Rabbits and partridges were the prey categories that most varied in terms of consumption among population-years, with Andalusia 2011 showing the highest mean consumption of these two prey, and Catalonia 2008 showing the lowest values (Table 2). Considerable variation in prey consumption was found among territories within the same population in a given year both in France or Catalonia, especially in the consumption of rabbits, partridges, domestic pigeons from dovecotes and squirrels. On the other hand, territories in Andalusia showed more homogeneous diets, with higher variation in the consumption of partridges than rabbits (Table 2).

**Table 2:** Mean and range (maximum-minimum) prey consumption (%) of Bonelli’s eagle nestlings on each monitored population-year calculated from the dietary estimates at the territory-year level obtained from Bayesian isotopic mixing models (SIAR).

Population	France	France	Catalonia	Catalonia	Catalonia	Catalonia	Andalusia
Year	2010	2011	2008	2009	2010	2011	2011
Prey category	Mean (range) %	Mean (range) %	Mean (range) %	Mean (range) %	Mean (range) %	Mean (range) %	Mean (range) %
OC	26.9 (34.5)	23.4 (22.5)	21.9 (36.6)	23.4 (49.1)	27.4 (52.3)	28.2 (45.6)	29.6 (16.7)
AR	20.4 (27.2)	17.5 (26.0)	15.2 (12.1)	17.2 (21.7)	19.0 (21.8)	17.6 (17.3)	24.6 (24.1)
CP	10.2 (08.3)	11.3 (07.2)	12.0 (06.3)	11.3 (07.2)	11.0 (09.7)	11.0 (08.4)	09.6 (8.7)
CLw	11.8 (06.6)	12.0 (14.2)	11.4 (07.9)	12.3 (15.5)	12.2 (21.5)	11.1 (11.7)	12.7 (4.7)
CLd	05.1 (19.1)	05.4 (13.2)	06.4 (16.9)	05.8 (16.4)	04.2 (13.4)	04.4 (13.0)	02.8 (3.3)
PAS	08.3 (09.4)	08.7 (8.2)	10.0 (09.5)	09.2 (11.3)	08.9 (12.8)	08.6 (08.3)	07.3 (8.2)
SV	06.8 (11.8)	08.4 (14.9)	09.6 (16.0)	08.3 (14.1)	06.0 (16.0)	07.0 (11.8)	04.2 (5.4)
TL	10.5 (10.5)	13.2 (10.8)	13.5 (07.0)	12.5 (09.4)	11.4 (10.4)	12.1 (10.5)	09.3 (9.4)

Note: OC = European rabbits; AR = red-legged partridges; CP = wood pigeons; CLw = domestic pigeons wildly foraging in crops; CLd = domestic pigeons from dovecotes and fed with corn; PAS = passerines; SV = Eurasian red squirrels; TL = ocellated lizards.

At the territory level, H′ was strongly negatively correlated with the consumption of rabbits (*r_s_* = −0.92, *P*<0.001) and partridges (*r_s_* = −0.82, *P*<0.001; Figure S1), but positively correlated with the consumption of wood pigeons (*r_s_* = 0.90, *P*<0.001), domestic pigeons from dovecotes (*r_s_* = 0.95, *P*<0.001), passerines (*r_s_* = 0.89, *P*<0.001), squirrels (*r_s_* = 0.90, *P*<0.001), and ocellated lizards (*r_s_* = 0.81, *P*<0.001). We did not find a significant linear correlation between H′ and PS_i_ (*r_s_* = 0.205, *P*>0.05). Nevertheless, we found that the mean population diet (i.e. highest PS_i_) coincided with intermediate H′ values, so either higher H′ (i.e. more generalized diets) or lower H′ (i.e. more specialized diets) reduced PS_i_ values (see Figure S2).

### Influence of Nestling Diet on Productivity

**Figure 4:**
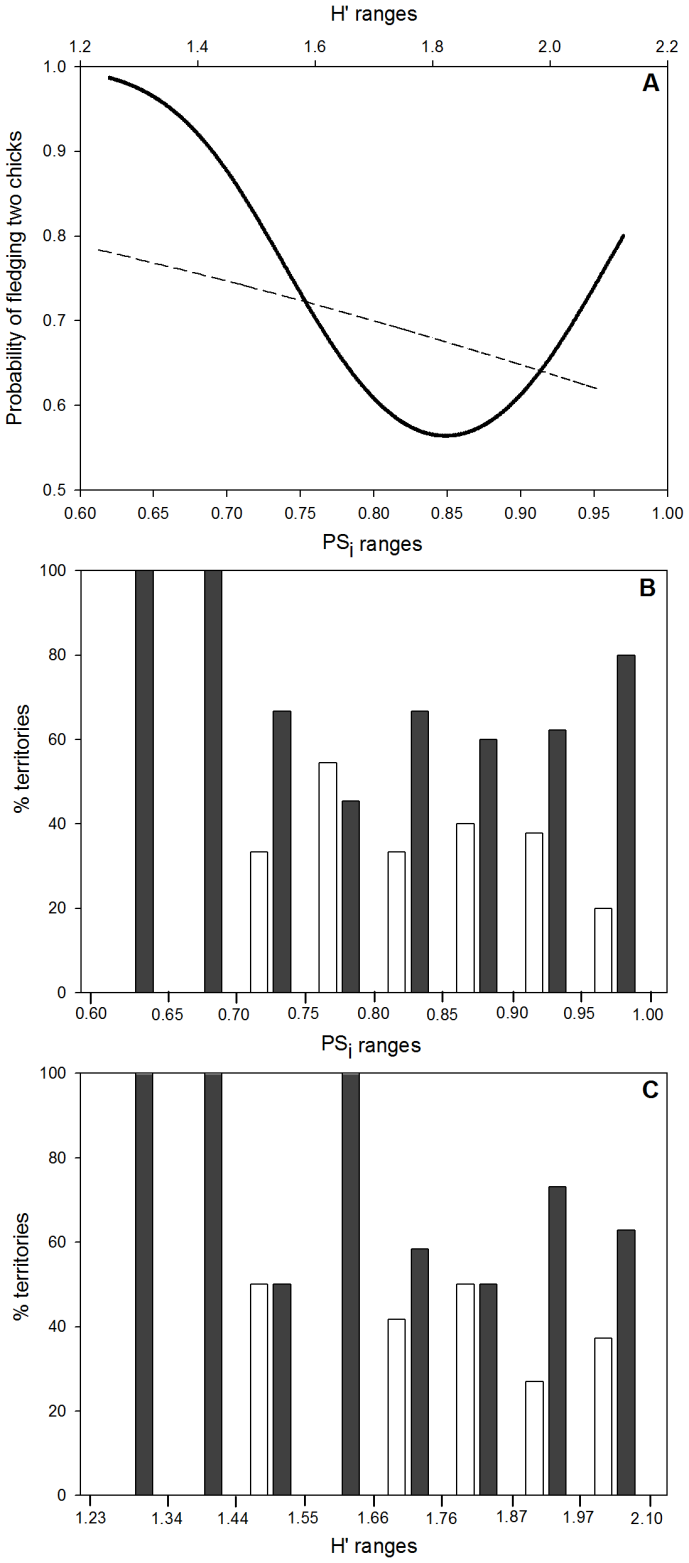
Responses of the second and third best models based on the GLMM approach. The productivity of successful breeding pairs (i.e. probability of producing two chicks instead of one) was the response variable, and age of breeding pairs, individual replacement, nestling prey consumption specificity (PS_i_) or diet diversity (H′) are the explanatory variables. The graphic (A) represents the modeled probability of fledging two chicks (y-axis, probabilities between 0.5 and 1.0) if only considering adult pairs with no individual replacement, and using the range of observed PS_i_ values (solid line and lower x-axis, PS_i_ between 0.62 and 0.97) or H′ values (dashed line and upper x-axis, H′ between 1.23 and 2.08). The graphics (B and C) shows the real data used in the GLMM, with the x-axis representing PS_i_ (B) or H′ (C) values of each territory, and the y-axis the proportion of territories fledging one (light bars) or two (dark bars) chicks.

The GLMMs showed that the productivity at the territory-year level was best explained by the age of pair and mate replacement, and by the age of pair and mate replacement together with the quadratic effect of PS_i_ or the negative effect of H′ (Table 3, Table S3). That is, either adult breeding pairs feeding their chicks with similar diets to the overall population (highest PS_i_ values and intermediate H′) or pairs disproportionally exploiting a single or few preferred prey types (lowest values of both PS_i_ and H′) were more likely to fledge two chicks than pairs with nestling showing intermediate PS_i_ values and higher H′ (see Figures 4 and S2). Nevertheless, the coefficient of determination of best fitted models indicated that the models have in general rather low explanatory power (see Table 3).

**Table 3:** Model selection to assess the effects of age, mate replacements and diet on the productivity of breeding successful pairs.

Model definition	ΔAIC_c_	AIC_cw_	R^2^_GLMM(m)_	R^2^_GLMM(c)_
**1. Age+Replacement**	**0.000** *	**0.215**	0.036	0.109
**2. Age+Replacement+PS_i_+PS_i_^2^**	**0.301**	**0.185**	0.107	0.206
**3 Age+Replacement+H′**	**1.774**	**0.088**	0.042	0.114
4. Age+Replacement+(OC+AR)	2.074	0.076	0.038	0.111
5. Age+Replacement+OC	2.129	0.074	0.037	0.105
6. Age+Replacement+PS_i_	2.149	0.073	0.037	0.108
7. Age+Replacement+AR	2.173	0.072	0.037	0.119
8. Age+Replacement+H′+H′^2^	3.331	0.041	0.058	0.118
9. Age+Replacement+(OC+AR)+H′	3.693	0.034	0.048	0.122
10. Age+Replacement+OC+H′	3.852	0.031	0.045	0.132
11. Age+Replacement+AR+H′	4.078	0.028	0.042	0.110
12. Age+Replacement+(OC+AR)+PS_i_	4.320	0.025	0.039	0.109
13. Age+Replacement+OC+PS_i_	4.362	0.024	0.038	0.104
14. Age+Replacement+AR+PS_i_	4.399	0.024	0.038	0.115
15. Age+Replacement+H′+PS_i_+(H′*PS_i_)	6.185	0.010	0.047	0.120

Model definition enumerates the fixed effects considered in the GLMMs. All models included territory and year nested by population as random effects.

*Best model AIC_c_ **= **174.899.

Note: OC = rabbit consumption; AR = partridge consumption; H′ = diet diversity; PS_i_ = prey consumption specificity. Parameters’ interactions are denoted by (*), while (^2^) indicates a quadratic effect. ΔAIC_c_ refers to the difference in the corrected Akaike Information Criteria (AIC_c_) between model *i* and the model with the lowest (AIC_c_) (i.e. the best model). Models with ΔAIC_c_ <2 are shown in bold type. The Akaike weights (AIC_cw_) explains the probability that a given candidate model is the best of the proposed set, so the sum of all the models is 1.0. R^2^_GLMM(m)_ estimates model fit using fixed effects only, while R^2^_GLMM(c)_ estimates model fit including both fixed and random effects.

**Figure 5:**
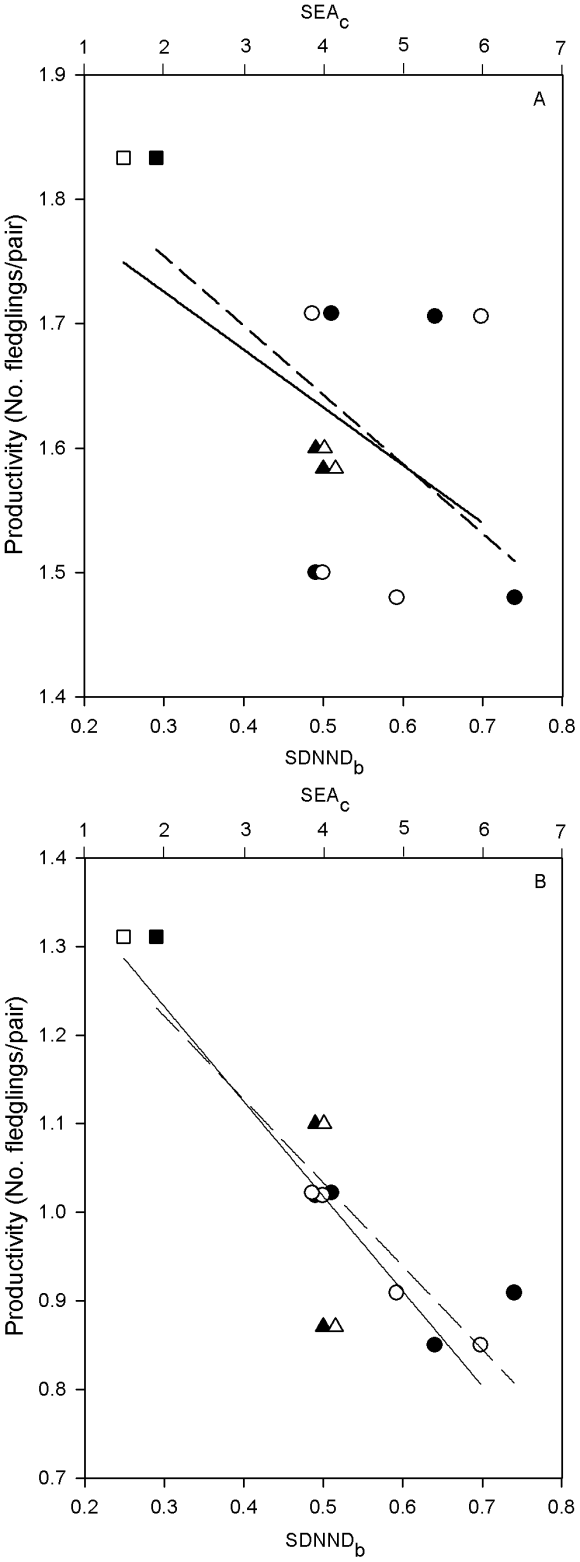
Correlations between mean productivity and both the SEA_c_ and the SDNND_b_ at the population-year level. Mean productivity refers to A) only successful pairs and B) the whole monitored population (either successful or not successful breeding pairs). In both cases, the upper x-axis shows the SEA_c_ (corrected standard ellipse area; open symbols), and the lower x-axis shows the SDNND_b_ (standard deviation of nearest neighbour distance bootstrapped; filled symbols). Trend lines of the relationship between productivity and either SEA_c_ (solid line) or SDNND_b_ (dashed line) are shown. Different symbol shapes represent France (triangles), Catalonia (circles) and Andalusia (squares).

At the population-year level, mean productivity of successful pairs were not correlated neither with SEA_c_ (*r_s_* = −0.54, *P* = 0.215) nor with SDNND_b_ (*r_s_* = −0.40, *P* = 0.379). We found, however, a significant and negative correlation between mean productivity in the whole population-year and the SEA_c_ (*r_s_* = −0.86, *P* = 0.014), and a marginally significant negative correlation between mean productivity and the SDNND_b_ (*r_s_* = −0.72, *P* = 0.068), suggesting that those population-years with more heterogeneous territories in terms of diet were less productive than population-years with territories showing more homogeneous prey consumption patterns (Figure 5).

## Discussion

Inter-individual diet variation is a widespread phenomenon within animal populations, but traditionally underappreciated [8]. Theory and empirical evidence of its ecological causes and eco-evolutionary consequences have been intensively addressed in recent years [8], [10], [19], [27]. In the case of terrestrial territorial predators, spatial differences in the abundance and availability of prey have been highlighted as an important factor influencing individual (e.g. territorial) diet differences [21], [24], but its fitness consequences to avian predators inhabiting terrestrial complex ecosystems like the Mediterranean region are poorly known. In this study, we used an isotopic approach to assess inter-individual (i.e. inter-territorial) diet variation within three Bonelli’s eagle populations of western Europe, and how different prey consumption patterns affected both the territory and the population breeding performance.

The use of SIA to assess the trophic niche width and individual resource partitioning within and among populations has proved a powerful tool in recent years [31], [32], [34], [35]. The sensitivity analysis on the TDFs supported our dietary results at the territory level, which, overall, highlighted diet variation among territories in the French and Catalonian populations (the northernmost populations of Bonelli’s eagle in Europe), while territories in the Andalusian population showed more homogeneous diets (Table 2). Differences in the intra-population diet variation could be explained by higher heterogeneity in prey availability among French and Catalonian territories compared with those in Andalusia, especially in terms of rabbits, partridges, domestic pigeons from dovecotes or squirrels, which were the most variable prey in the diet (Table 2). We also found a significant negative correlation between territorial consumption of rabbits or partridges and H′, suggesting these prey are optimal for Bonelli’s eagle in the study area [3], [6] as found in previous studies that used traditional diet examination methods [46], [48], [49]. Thus, rabbits and partridges could be positively selected by Bonelli’s eagle when available, reducing the diet diversity, while higher consumption of other prey like pigeons, other birds, squirrels or lizards could compensate a shortage of the former prey, especially in some territories in France and Catalonia (see [48], [49], [66]). The challenge then was to assess whether differences in prey consumption at both the territory and population levels could affect breeding performance.

The fitness consequences of individual diet variation are the result of a complex interplay between individual foraging behavior and abilities, variation in resource preferences, and heterogeneity in resource availability. Thus, different feeding strategies may be advantageous for different species and/or ecological scenarios, an issue mostly addressed in some colonial seabirds [16], [17], [19]. Consumers may increase their breeding success by specializing when capturing prey for their chicks, probably because they become more efficient foragers [16]. Conversely, differences in reproductive success between specialists and generalists not always emerge, presumably because both foraging strategies can be advantageous at different levels of prey abundance or predictability [19]. Nevertheless, studies on terrestrial territorial avian predators have been scarce (but see [20], [21], [24]), probably due to the difficulty in monitoring predator’s main vital rates and their dietary patterns on large spatial scales.

At the territory level, Bonelli’s eagle productivity was higher in those territories showing few individual turnover rates, as expected in long-lived species [61], [62], and according with the fact that among birds, reproductive success often increases with age [67]. The lower productivity we found in non-adult breeding pairs could be related with their intrinsic lower quality (e.g. breeding inexperience, lower foraging efficiency or competitive ability) (see [61], [68]). Diet had also an effect on the productivity of breeding successful pairs, and both prey consumption specificity (PS_i_) and diet diversity (H′) allowed predicting the number of chicks fledged (Figure 4). Pairs disproportionally exploiting a single or few preferred prey types (lowest values of PS_i_ and H′) were more likely to fledge two chicks. Nevertheless, there were few territories where nestlings disproportionally consumed rabbits and/or partridges, suggesting that the scenario of superabundance of preferred prey is rare in the study area (see [48]). Furthermore, pairs whose nestlings consumed prey in similar proportions than the overall population (highest PS_i_ values and intermediate H′) were also more likely to fledge two chicks. In this case, mean population diet included moderate consumption of rabbits, pigeons and partridges, which possibly corresponds to the more common scenario in suitable habitats for the Bonelli’s eagle in our study area. Our results thus suggest that Bonelli’s eagles may benefit in terms of productivity either from high consumption of preferred prey like rabbits and/or partridges, but also from moderate intake of these prey provided that they are abundantly complemented by some key alternative prey, such as pigeons. High values of diet diversity, however, had a negative effect on productivity, possibly as a consequence of higher consumption of a variety of suboptimal prey triggered by the scarcity of preferred prey (see [5]). It is worth to mention that our best fitted models had low explanatory power. This fact could be related to low variation in diet composition due to the exclusion of data from unsuccessful pairs. Nevertheless, other causes apart from diet may have great impact on productivity (e.g. human disturbances, inter-specific competition, etc.), which can difficult our ability to detect the diet effects on productivity. Additionally, we do not discard that high turnover rates may generate covariation between diet diversity and productivity (see [69]).

At the population-year level, we found a negative correlation between mean productivity and the SEA_c_ (Figure 5), suggesting a link between higher productivity and lower trophic niche width. The productivity of successful pairs, however, was not correlated with any isotopic niche metric, a finding that can be explained because temporal variance in productivity of successful pairs is very low in our study area (unpublished data). For instance, in Catalonia there were poor environmental conditions (e.g. cold and rainy days) in spring 2009 that negatively affected the productivity of Bonelli’s eagle, which was remarkably low (0.85, n = 40), while the productivity of successful pairs on that breeding season (1.71, n = 17) was similar or even higher than in other years. Apparently, pairs holding good territories are able to rear chicks even in bad years [70], but this does not necessarily implies that the environmental and food supply conditions that these pairs experience are the same over years, as suggested by our results. In particular, the population-year with the highest mean productivity (i.e. Andalusia 2011) showed the lowest SEA_c_, while the population-year with the lowest mean productivity (i.e. Catalonia 2009) showed the highest SEA_c_. In the case of Andalusia, lower SEA_c_ was concordant with an overall higher consumption of preferred prey, which ultimately could have increased mean productivity [29], [71]. Assuming that consumers’ diet diversity usually increase as food becomes limiting [5], our results suggest higher heterogeneity in preferred prey availability among territories in France or Catalonia compared with Andalusia. In contrast, higher consumptions of preferred prey at the population level increased diet homogeneity among territories, as we found in the case of Andalusia that showed the lowest SDNND_b_ value. Indeed, this population shows the highest values of main vital rates over the western European range of Bonelli’s eagle [43]. Differences in individual’s foraging abilities may also influence diet variation [8]. Nonetheless, inter-individual differences in foraging behaviour were expected to be similar in the three populations so we did not expect that this effect may explain the suggested differences in main dietary patterns of France and Catalonia compared with Andalusia, which are indeed inter-connected each other through dispersal processes [43]. Therefore, we concur with [21] that within population diet variation in our case study could primarily be a consequence of variation in prey availability among territories. However, variation in productivity could also arise from differences in the percentage of non-adult pairs among populations. In this regard, Andalusia holds the larger mean percentage of adult pairs and Catalonia the lowest (see “Study area” in “Materials and Methods”). Thus, age of breeders, turnover rates and territory quality could simultaneously affect reproductive output within a population (as suggested by our previous results at the territory level), and ultimately drive productivity at the population level (see [61], [69], [72], [73]).

Our study has relevant conservation implications because Bonelli’s eagle is a threatened raptor in Mediterranean countries, and is listed as “endangered” in Europe [74]. We suggest that SIA could be a useful tool to monitor the species’ diet on large spatio-temporal scales to detect potential changes in the main prey on which Bonelli’s eagle depends for breeding. In some Bonelli’s eagle populations, the low productivity prevents an adequate demographic balance and recruitment of birds [43]. Thus, improving the availability of optimal prey as European rabbit and red-legged partridge in certain Bonelli’s eagle territories can be an important conservation tool to enhance their viability. Increasing the populations of alternative prey such as pigeons should be also considered to compensate the potential shortage of preferred prey, especially in highly degraded environments and where rabbit haemorrhagic disease has drastically depleted rabbit abundances (see [48], [66]).

The rapid development of quantitative analytical approaches for applying stable isotope data in studies of individual animal foraging ecology offers a new perspective to test hypotheses under the framework of the optimal foraging theory [2], [3]. While dietary information at the individual level is difficult to obtain for large avian predator species by conventional diet analysis, isotopic derived metrics based on both δ- and p-space can be used to address diet variation at the intra-population level, as well as its eco-evolutionary consequences (see [8], [25]). Nevertheless, the application of stable isotope analyses to assess nestling trophic ecology is obviously limited to successful breeding territories. A solution to explore diet-fitness relationships at the territory level could be to study the diet of parents instead of nestlings, but field sampling effort (e.g. blood samples for isotopic analysis, pellet collection, etc.) would notably increase. Another shortcoming of using stable isotope analyses was that they do not provide information on biomass consumed, which may also influence productivity. In our case, however, it has been described a direct correspondence in both the qualitative and quantitative representation of the different prey groups in the diet of the Bonelli’s eagle (at least in part of our study area, [75]), which support our general conclusions. Overall, in this study we have illustrated that, despite limitations, monitoring diet and fitness of individuals and populations distributed over large geographical ranges has indeed a great potential to further understand the fitness consequences of variation in resource use within- and between-populations. Also, we have shown the success of this approach in complex terrestrial ecosystems like the Mediterranean, where the number of potential confounding factors is greater than in other predator-prey systems [49]. Thus, we encourage the use of similar approaches to ecologists as well as evolutionary and conservation biologists concerned with the multi-scale fitness consequences (not only breeding success, but also other indicators such as body condition or survival) of inter-individual variation in resource use.

## Supporting Information

Figure S1 **Relationship between the diet diversity (H′) and the consumption percentage (%) of rabbits (A) and partridges (B) at the territory level (** ***n*** **= 71).**
(DOC)

Figure S2 **Relationship between the diet diversity (H′) and the prey consumption specificity (PS_i_) at the territory level (** ***n*** **= 71).**
(DOC)

Table S1 Mean ± SD (‰) values of δ^13^C and δ^15^N in the Bonelli’s eagle prey categories included in SIAR.
(DOC)

Table S2 Explanatory variables used in the GLMMs to assess their potential effect on Bonelli’s eagle productivity, classified either as spatiotemporal parameters, breeding pair parameters or diet parameters.
(DOC)

Table S3 Summary of model parameter estimates and standard error of parameter estimates for each model included in the GLMMs. Models are showed following the same order as in Table 3.
(DOC)
