# Supplementary material for: Multi-Scale Effects of Nestling Diet on Breeding Performance in a Terrestrial Top Predator Inferred from Stable Isotope Analysis
Source: PLoS One. 2014 Apr 17;9(4):e95320. doi: 10.1371/journal.pone.0095320 (PMC3990674; doi:10.1371/journal.pone.0095320)

**Figure S1.** **Relationship between the diet diversity (H’) and the consumption percentage (%) of rabbits (A) and partridges (B) at the territory level (*n* = 71).**


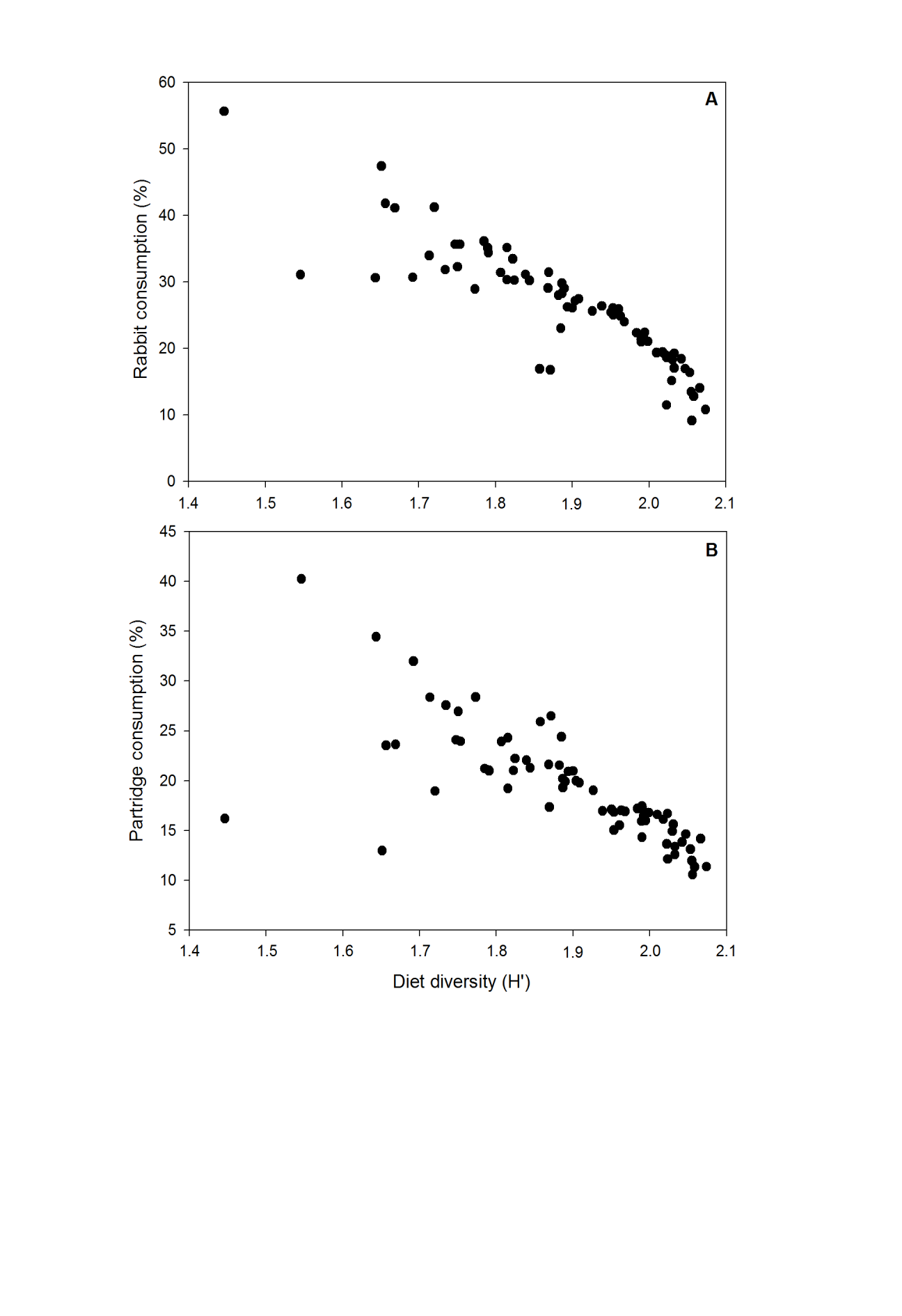

Supplement: Figure S1 — Relationship between the diet diversity (H′) and the consumption percentage (%) of rabbits (A) and partridges (B) at the territory level ( n = 71). (DOC) [file pone.0095320.s001.doc]
