# Supplementary material for: Multi-Scale Effects of Nestling Diet on Breeding Performance in a Terrestrial Top Predator Inferred from Stable Isotope Analysis
Source: PLoS One. 2014 Apr 17;9(4):e95320. doi: 10.1371/journal.pone.0095320 (PMC3990674; doi:10.1371/journal.pone.0095320)

**Figure S2. Relationship between the diet diversity (H’) and the prey consumption specificity (PS_i_) at the territory level (*n* = 71).**


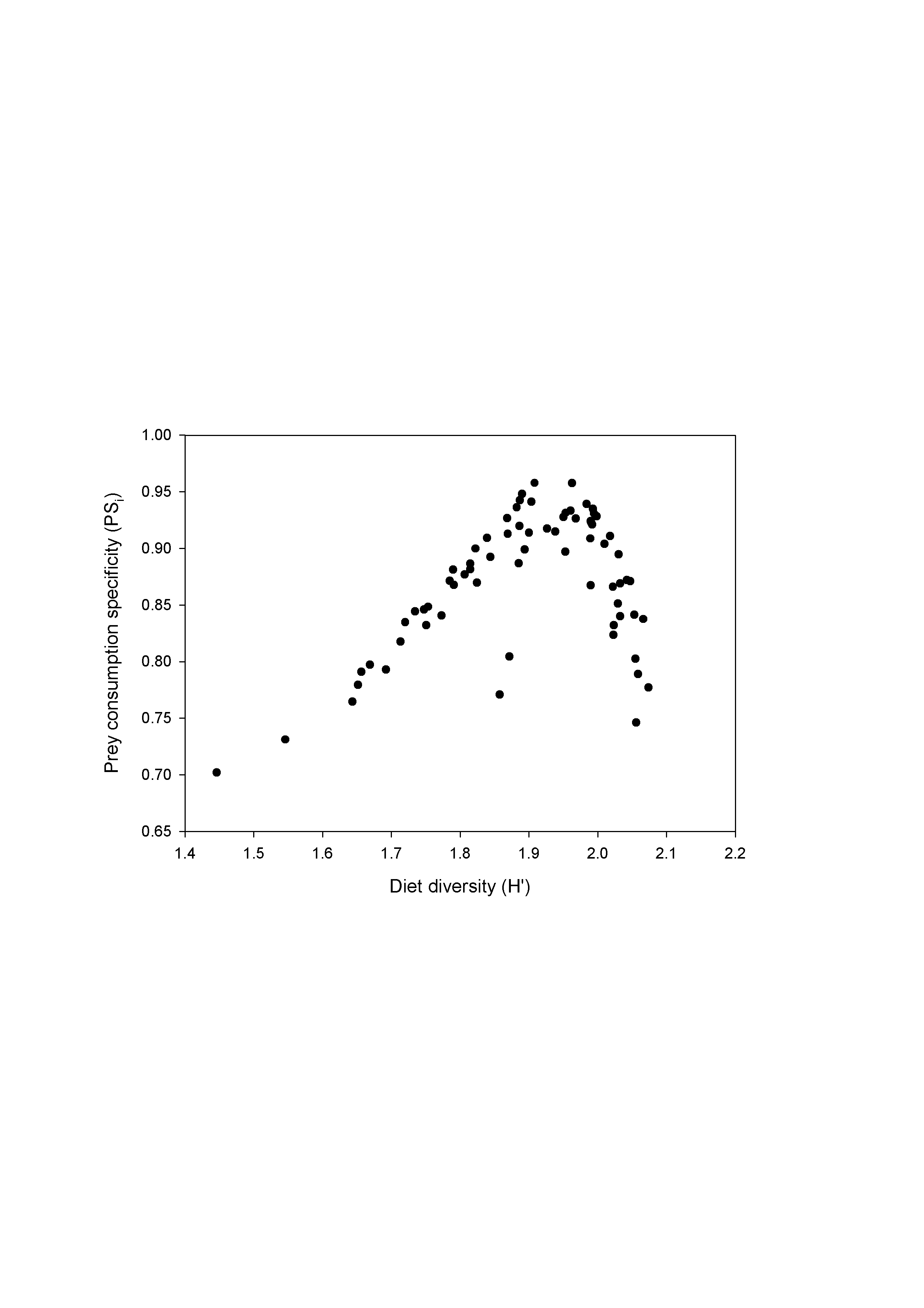

Supplement: Figure S2 — Relationship between the diet diversity (H′) and the prey consumption specificity (PSi) at the territory level ( n = 71). (DOC) [file pone.0095320.s002.doc]
