# Supplementary material for: Multi-Scale Effects of Nestling Diet on Breeding Performance in a Terrestrial Top Predator Inferred from Stable Isotope Analysis
Source: PLoS One. 2014 Apr 17;9(4):e95320. doi: 10.1371/journal.pone.0095320 (PMC3990674; doi:10.1371/journal.pone.0095320)
Supplement: Table S1 — Mean ± SD (‰) values of δ13C and δ15N in the Bonelli’s eagle prey categories included in SIAR. (DOC) [file pone.0095320.s003.doc]

**Table S1.** **Mean ± SD (‰) values of δ^13^C and δ^15^N in the Bonelli’s eagle prey categories included in SIAR.**

| **Prey category** | **δ^13^C Mean ± SD (‰)** | **δ^15^N Mean ± SD (‰)** | ***n*** |
| --- | --- | --- | --- |
| OC | -26.58 ± 0.43 | 2.81 ± 1.90 | 14 |
| AR | -25.60 ± 0.96 | 5.48 ± 2.12 | 10 |
| CP | -23.92 ± 0.79 | 5.52 ± 1.70 | 42 |
| CLw | -23.64 ± 1.42 | 6.73 ± 2.23 | 41 |
| CLd | -16.75 ± 2.17 | 6.85 ± 1.19 | 19 |
| PAS | -23.36 ± 0.72 | 7.25 ± 1.24 | 40 |
| SV | -19.36 ± 1.60 | 3.23 **±** 1.99 | 7 |
| TL | -24.04 ± 0.28 | 4.50 ± 2.38 | 3 |

Note: OC = European rabbits; AR = red-legged partridges; CP = wood pigeons; CLw = domestic pigeons wildly foraging in crops; CLd = domestic pigeons from dovecotes and fed with corn; PAS = passerines; SV = Eurasian red squirrels; TL = ocellated lizards; n = number of individuals analysed for each prey category.
