# Supplementary material for: Multi-Scale Effects of Nestling Diet on Breeding Performance in a Terrestrial Top Predator Inferred from Stable Isotope Analysis
Source: PLoS One. 2014 Apr 17;9(4):e95320. doi: 10.1371/journal.pone.0095320 (PMC3990674; doi:10.1371/journal.pone.0095320)
Supplement: Table S3 — Summary of model parameter estimates and standard error of parameter estimates for each model included in the GLMMs. Models are showed following the same order as in Table 3. (DOC) [file pone.0095320.s005.doc]

**Table S3. Summary of model parameter estimates and standard error of parameter estimates for each model included in the GLMMs. Models are showed following the same order as in Table 3.**

| Model 1 <- Age + Replacement + (1|Terr) + (Popu|Year) | | |
| --- | --- | --- |
| **Parameter** | **Estimate** | **Standard Error** |
| Intercept | 0.663 | 0.218 |
| Age | -1.067 | 0.860 |
| Replacement | -0.142 | 0.807 |
| Model 2 <- Age + Replacement + PS_i_ + (PS_i_^2^) + (1|Terr) + (Popu|Year) | | |
| **Parameter** | **Estimate** | **Standard Error** |
| Intercept | 55.660 | 29.170 |
| Age | -1.116 | 0.884 |
| Replacement | -0.008 | 0.831 |
| PS_i_ | -130.500 | 68.970 |
| PS_i_^2^ | 76.890 | 40.580 |
| Model 3 <- Age + Replacement + H’ + (1|Terr) + (Popu|Year) | | |
| **Parameter** | **Estimate** | **Standard Error** |
| Intercept | 2.448 | 2.486 |
| Age | -1.045 | 0.861 |
| Replacement | -0.176 | 0.808 |
| H’ | -0.942 | 1.304 |
| Model 4 <- Age + Replacement + (OC+AR) + (1|Terr) + (Popu|Year) | | |
| **Parameter** | **Estimate** | **Standard Error** |
| Intercept | 0.344 | 0.705 |
| Age | -1.053 | 0.860 |
| Replacement | -0.167 | 0.809 |
| OC+AR | 0.007 | 0.015 |
| Model 5 <- Age + Replacement + OC + (1|Terr) + (Popu|Year) | | |
| **Parameter** | **Estimate** | **Standard Error** |
| Intercept | 0.445 | 0.562 |
| Age | -1.037 | 0.859 |
| Replacement | -0.150 | 0.806 |
| OC | 0.008 | 0.020 |
| Model 6 <- Age + Replacement + PS_i_ + (1|Terr) + (Popu|Year) | | |
| **Parameter** | **Estimate** | **Standard Error** |
| Intercept | 1.549 | 2.343 |
| Age | -1.067 | 0.860 |
| Replacement | -0.146 | 0.806 |
| PS_i_ | -1.021 | 2.678 |
| Model 7 <- Age + Replacement + AR + (1|Terr) + (Popu|Year) | | |
| **Parameter** | **Estimate** | **Standard Error** |
| Intercept | 0.446 | 0.668 |
| Age | -1.087 | 0.866 |
| Replacement | -0.174 | 0.812 |
| AR | 0.012 | 0.034 |
| Model 8 <- Age + Replacement + H’ + (H’^2^) + (1|Terr) + (Popu|Year) | | |
| **Parameter** | **Estimate** | **Standard Error** |
| Intercept | 21.221 | 23.830 |
| Age | -1.076 | 0.858 |
| Replacement | -0.132 | 0.805 |
| H’ | -21.854 | 26.141 |
| H’^2^ | 5.769 | 7.139 |
| Model 9 <- Age + Replacement + (OC + AR) + H’ + (1|Terr) + (Popu|Year) | | |
| **Parameter** | **Estimate** | **Standard Error** |
| Intercept | 8.462 | 9.790 |
| Age | -1.043 | 0.863 |
| Replacement | -0.157 | 0.810 |
| OC+AR | -0.030 | 0.047 |
| H’ | -3.399 | 4.092 |
| Model 10 <- Age + Replacement + OC + H’ + (1|Terr) + (Popu|Year) | | |
| **Parameter** | **Estimate** | **Standard Error** |
| Intercept | 5.508 | 6.370 |
| Age | -1.097 | 0.874 |
| Replacement | -0.195 | 0.813 |
| OC | -0.023 | 0.043 |
| H’ | -2.237 | 2.813 |
| Model 11 <- Age + Replacement + AR + H’ + (1|Terr) + (Popu|Year) | | |
| **Parameter** | **Estimate** | **Standard Error** |
| Intercept | 2.917 | 3.859 |
| Age | -1.028 | 0.863 |
| Replacement | -0.163 | 0.808 |
| AR | -0.007 | 0.045 |
| H’ | -1.120 | 1.720 |
| Model 12 <- Age + Replacement + (OC + AR) + PS_i_ + (1|Terr) + (Popu|Year) | | |
| **Parameter** | **Estimate** | **Standard Error** |
| Intercept | 1.079 | 2.605 |
| Age | -1.055 | 0.860 |
| Replacement | -0.167 | 0.808 |
| OC+AR | 0.006 | 0.015 |
| PS_i_ | -0.802 | 2.731 |
| Model 13 <- Age + Replacement + OC + PS_i_ + (1|Terr) + (Popu|Year) | | |
| **Parameter** | **Estimate** | **Standard Error** |
| Intercept | 1.216 | 2.513 |
| Age | -1.041 | 0.860 |
| Replacement | -0.153 | 0.806 |
| OC | 0.007 | 0.020 |
| PS_i_ | -0.857 | 2.713 |
| Model 14 <- Age + Replacement + AR + PS_i_ + (1|Terr) + (Popu|Year) | | |
| **Parameter** | **Estimate** | **Standard Error** |
| Intercept | 1.259 | 2.550 |
| Age | -1.084 | 0.865 |
| Replacement | -0.172 | 0.810 |
| AR | 0.010 | 0.035 |
| PS_i_ | -0.897 | 2.718 |
| Model 15 <- Age + Replacement + H’ + PS_i_ + (H’ * PS_i_) + (1|Terr) + (Popu|Year) | | |
| **Parameter** | **Estimate** | **Standard Error** |
| Intercept | 15.116 | 25.294 |
| Age | -1.051 | 0.862 |
| Replacement | -0.167 | 0.809 |
| H’ | -7.692 | 13.508 |
| PSi | -16.259 | 32.107 |
| H’*PSi | 8.628 | 17.01 |

Note: models are ordered from higher to lower AIC_cw_ values following the classification and nomenclature shown in Table 3.
